# Growth Performance, Blood Chemistry, and Intestinal Bacterial Community of Florida Pompano (*Trachinotus carolinus*) Fed Different Levels of Corn Fermented Protein and Yeast Diets

**DOI:** 10.1155/anu/8872997

**Published:** 2025-08-22

**Authors:** Trinh H. V. Ngo, Marty Riche, Timothy J. Bruce, D. Allen Davis

**Affiliations:** ^1^School of Fisheries, Aquaculture and Aquatic Sciences, Auburn University, Auburn, Alabama, USA; ^2^Harbor Branch Oceanographic Institute, Florida Atlantic University, Fort Pierce, Florida, USA

**Keywords:** blood biochemistry, corn fermented protein, growth, gut microbiota, *Trachinotus carolinus*, yeast

## Abstract

Developing species-specific diets for Florida pompano (*Trachinotus carolinus*) requires understanding both nutritional needs and ingredient responses in practical formulations. Previous research has successfully reduced animal protein inclusion to ~15% by using solvent-extracted soybean meal (SBM) as the primary protein source. Further cost savings may be achieved by incorporating other low-cost alternatives, such as corn fermented protein (CFP), a new sustainable ingredient produced using Fluid Quip Technologies. A 12-week growth trial was conducted with juvenile Florida pompano (initial weight 6.08 ± 0.55 g) using a fishmeal-free basal diet. The basal diet contained poultry by-product meal (15% diet) and the SBM (52% diet) as primary protein sources. The SBM was then incrementally replaced with CFP (5%, 10%, and 20% diet) and *Saccharomyces cerevisiae* fermentation product (FSC) (2%) on an equal protein basis. All diets were formulated to be isonitrogenous (40% protein) and isolipidic (8% lipid), and fish were reared in a recirculating aquaculture system (RAS). Fish across all treatments exhibited similar growth metrics, including final weight (FW) (44.61–56.98 g), weight gain (WG) (557%–738%), and feed conversion ratio (FCR) (1.56–1.75), with no significant differences (*p*  > 0.05). Blood parameters remained within healthy ranges, and microbiome analyses revealed stable gut bacterial diversity and composition among treatments. These results suggest that CFP can be used as a protein source in practice for Florida pompano without impairing growth, health status, or intestinal microbiota composition.

## 1. Introduction

The Florida pompano, scientifically known as *Trachinotus carolinus*, is a species of warm-water, marine teleost fish belonging to the jack family *Carangidae*. The species is highly valued in the seafood market, but demand exceeds what can be supplied through catch fisheries alone [1]. Due to its rapid growth rate and adaptability to various environmental conditions, the Florida pompano has been successfully cultivated for sustainable and commercial aquaculture [2]. Its economic potential and high consumer demand highlight the need for research aimed at optimizing its growth performance and overall health. A crucial aspect of successful fish farming is understanding the dietary requirements of cultured fish, which necessitates the study of fish nutrition, including biotechnology, feed additives, and probiotics [3].

Fish meal (FM) has historically been the favored protein source in aquaculture feed formulations owing to its superior digestibility, palatability, and balanced amino acid (AA) composition. Supply limitations, high pricing, and environmental concerns have prompted efforts to replace FM with alternative sources. Plant-based feed ingredients have gained significant attention as sustainable alternatives to FM due to their availability, affordability, and reduced environmental impact [4]. Among these, soybean meal (SBM) has traditionally served as the primary protein source in feed due to its high protein content, balanced AA profile, and consistent quality [5]. However, the presence of antinutritional factors (ANFs) and nonstarch polysaccharides (NSPs) limits their utility in some species, particularly for young fish species [6]. For Florida pompano, soy protein isolate or solvent-extracted SBM and poultry by-product meal can partially or completely replace FM without affecting growth performance [7]. The results demonstrate the species' ability to adapt to diets free of FM and the viability of using sustainable feed ingredients.

Corn fermented protein (CFP) could be a significant alternative to conventional protein sources such as SBM and FM, especially in the United States, producing roughly one-third of the world's corn [8]. CFP is produced utilizing sophisticated methodologies, including the Maximized Stillage Coproducts (MSC) technology developed by Fluid Quip Technologies [9]. This technology separates premium nutrients, including protein, fiber, and yeast, from stillage, a coproduct of dry-grind ethanol manufacturing. The MSC procedure improves CFP's nutritional profile by reducing ANFs and guarantees a greater concentration of bioavailable AAs and minerals relative to conventional distillers dried grains with solubles (DDGSs) [10]. The extensive use of this technique in ethanol plants throughout the United States and South America has resulted in a product containing ~20%–25% yeast (on a dry matter basis), which has demonstrated superior digestibility and growth in aquaculture species [11]. Research has shown favorable outcomes with CFP in species such as Atlantic salmon (*Salmo salar*) [12] and channel catfish (*Ictalurus punctatus*) [13]. Despite its demonstrated potential, CFP is a relatively new product that is now being produced for commercial use. Furthermore, there is limited data, particularly on marine species. Given the increasing pressure to develop sustainable ingredients in fish diets, the exploration of novel protein sources is crucial for the continued expansion of marine aquaculture. Alternative protein sources that can support growth performance while reducing reliance on FM are essential for the long-term sustainability of fish culture. With its excellent nutrient content, affordability, and environmentally friendly production, CFP meets the growing need for sustainable ingredients in fish diets while reducing dependance on more expensive and less available options.

Yeast and yeast-based products have gained popularity as a source of nutrients and bioactive compounds. Among these, fermentation products such as *Saccharomyces cerevisiae* fermentation product (FSC) have emerged as significant feed additives in aquaculture [14]. FSC represents a novel natural fermented product comprising yeast cell walls, specifically β-glucans and mannans, in addition to cell-soluble materials, and sporadically, living cells [15]. Studies have demonstrated that FSC can enhance growth performance, feed efficiency [16, 17], and potentially improve immune responses in several fish species [18–20]. It is a potential ingredient in aquaculture, improving growth, disease resistance, water quality, and fish immune systems, as noted by Sharma et al. [21].

Although both CFP (corn fermentation protein, comprising corn protein and yeast) and FSC, a yeast-derived supplement have shown potential advantages in a few species, their impacts on Florida pompano (*Trachinotus carolinus*) have yet to be investigated. Since FSC only reflects the yeast component, its inclusion in this study provides a direct comparison to separate and assess the distinct contributions of corn protein within CFP. By comparing the two, this study aimed to clarify the potential of corn protein as a protein source independent of the effects of yeast. Therefore, the objective of this research was to determine the impact of CFP and FSC on the growth performance, blood chemistry, and intestinal microbiota of Florida pompano, providing insights into the efficacy of CFP as a nutritional component in the aquaculture industry.

## 2. Materials and Methodology

### 2.1. Experimental Diets

All five experimental diets including 0%, 5%, 10%, 20% of CFP, and 2% of FSC were formulated to be isonitrogenous (40% protein) and isolipidic (8% lipid) and produced at the Aquatic Animal Nutrition Laboratory at the School of Fisheries, Aquaculture, and Aquatic Sciences, Auburn University (Auburn, Alabama, USA) using standard procedures for Florida pompano [22, 23]. The 2% inclusion of FSC was selected to approximate the yeast content in the CFP [11] and aligns with studies reporting benefits of 2% yeast supplementation in fish diets [24–27]. Briefly, diets were prepared by mixing the preground dry ingredients and oils in a food mixer (Hobart Corporation, Troy, Ohio, USA) for 10–15 min. Boiling water was then added to the mixture to obtain a consistency appropriate for pelleting. Diets were then pressure-pelleted using a meat grinder with a 3 mm die plate, and the wet pellets were then placed into a forced-air drying oven (<45°C) for ~24 h until the moisture content of each diet was less than 10%. A portion of each diet was crumbled and sieved prior to use in the early stages of the study, until the fish were large enough to handle a 3 mm pellet. All dried diets were stored in a freezer at −20°C until used in the experiment. The proximate composition and AA profile of the diets were analyzed at the University of Missouri Agricultural Experiment Station Chemical Laboratories (Columbia, Missouri, USA) and summarized in Tables 1 and 2, respectively.

### 2.2. Growth Trial

Florida pompano juveniles (~1 g) were purchased from Proaquatix LLC (Vero Beach, Florida, USA), a commercial hatchery with a disease-free status. All fish originated from a single production batch, were not genetically modified, and were considered immunocompetent at the start of the trial. No prior experimental procedures or treatments had been conducted on the fish before the study. Upon arrival, fish were nursed in an indoor recirculating aquaculture system (RAS) at the School of Fisheries, Aquaculture, and Aquatic Sciences, Auburn University (Auburn, Alabama, USA). During the nursery phase, fish were fed to apparent satiation with 1.5 mm commercial feed (FF Starter, Zeigler Bros. Inc., Gardner, Pennsylvania, USA), which contained 55% crude protein and 15% crude fat, until they reached an adequate size. The trial was conducted in a recirculating system (30 tanks) consisting of 120 L aquaria connected to a sump (800 L), bead filters (0.2 m^2^ media, 0.6 m × 1.1 m), a 0.25 hp circulation pump, supplemental aeration using a regenerative blower and air diffusers. Additionally, a small cartridge filter (Pentair plc, Golden Valley, Minnesota, USA) was supplemented to the system to remove any particulates that might contribute to water quality issues. At the start of the experiment, 10 size-sorted fish with a mean initial weight of 6.08 ± 0.55 g were stocked into each tank and assigned to six replicate tanks in a completely randomized design. The stocking density of 10 fish per 120 L aquarium was chosen to minimize stress and maintain optimal water quality, as Florida pompano is sensitive to high stocking densities [28]. Fish were maintained under a natural photoperiod with slight seasonal variation for 12 weeks. Routine system maintenance includes partial water exchanges, backwashing, adding sodium bicarbonate to maintain alkalinity and pH, and siphoning solid waste from the central reservoir as needed. Fish were hand-fed six times daily on a percentage of body weight basis (ranging from 5%–10%). Daily feeding levels were adjusted weekly based on growth and feeding response observation and feed inputs were calculated on a 2-week basis after sampling to adjust for growth and mortalities. To limit the possibility of parasite infection, fish were dipped in chloroquine phosphate (MP Biomedicals, Solon, Ohio, USA) as a bactericide at a concentration of 60 mg L^−1^, followed by a freshwater dip for approximately one to two minutes during sampling [29]. This prophylactic treatment was repeated every time fish were handled.

At the end of the trial, after an overnight fast, the fish groups were weighed, individuals counted, and numbers were determined to calculate the final weight (FW), weight gain (WG), feed conversion ratio (FCR), survival, thermal-unit growth coefficient (TGC), apparent net protein retention (ANPR), and energy retention (ER). Three fish per tank were randomly collected, individually weighed, and anesthetized with 100 mg L^−1^ tricaine methanesulfonate (MS-222, Western Chemical, Inc., Ferndale, Washington, USA). Blood samples were collected from the caudal vein of anesthetized fish using a 1 mL syringe with a 25-gauge needle (Exelint International, Redondo Beach, California, USA) into 1.5 mL eppendorf anticoagulant-free for serum biochemistry analysis and heparinized soda-lime glass micro-hematocrit capillary tubes (DWK Life Sciences LLC, Millville, New Jersey, USA), wax-sealed (Paul Marienfeld GmbH & Co. KG, Lauda-Königshofen, Germany) for hematocrit analysis. Furthermore, these same fish were then euthanized with buffered MS-222 for gut, liver collection, and weighed to calculate the hepatosomatic index (HSI). This study complies with the arrive guidelines for reporting animal research (Supporting Information 1: Arrive Checklist).

The following equations were used to calculate each growth parameter:

  $$\begin{array}{c} \text{Final weight }\left(\text{FW};g\right)\;=\text{Total biomass }\left(g\right)/\text{Final fish number}, \end{array}$$

  $$\begin{array}{c} \text{Weight gain }\left(\text{WG};\text{ \% }\right)\;=\text{Final weight }\left(g\right)\;-\text{Initial weight }\left(g\right)/\text{Initial weight }\left(g\right)\;\times \;100, \end{array}$$

  $$\begin{array}{c} \text{Survival }\left(\text{\% }\right)=\left(\text{Final fish number}/\text{Initial fish number}\right)\;\times \;100, \end{array}$$

  $$\begin{array}{c} \text{Feed conversion ratio }\left(\text{FCR}\right)=\text{Feed intake }\left(\text{dry weight }\left(g\right)\right)\;/\text{Weight gain }\left(\text{wet weight }\left(g\right)\right), \end{array}$$

  $$\begin{array}{c} \text{Thermal-unit growth coefficient }\left(\text{TGC}\right)\;=\;\left({\text{Final weight}}^{1/3}\;-{\text{Initial weight}}^{1/3}\right)/\;\left(\text{Water temperature }\left({}^{\circ}C\right)\times \text{Days}\right)\times 100, \end{array}$$

  $$\begin{array}{c} \text{ANPR }\left(\text{\% }\right)=\left(\text{FW }\left(g\right)\times \text{Final protein content }\left(\text{\% }\right)\right)\;-\;\left(\text{IW }\left(g\right)\times \text{Initial protein content }\left(\text{\% }\right)\right)/\text{Protein intake }\left(g\right)\times 100, \end{array}$$

  $$\begin{array}{c} \text{Energy retention }\left(\text{ER};\text{ \% }\right)\;=\;\left(\text{FW}\left(g\right)\times \text{Final energy content }\left(\text{kJ}/g\right)\right)\;-\;\left(\text{IW }\left(g\right)\times \text{Initial energy content }\left(\text{kJ}/g\right)\right)/\text{Energy intake }\left(g\right)\times 100, \end{array}$$

  $$\begin{array}{c} \text{Hepatosomatic index }\left(\text{HSI};\text{ \% }\right)\;=\text{Weight of liver }\left(g\right)/\text{Body weight }\left(g\right)\times 100. \end{array}$$

### 2.3. Water Quality

During the trial, the saltwater was maintained at around 11 g L^−1^ by combining artificial crystal sea salt (Crystal Sea Marinemix, Marine Enterprises International, Baltimore, Maryland, USA) with freshwater. Dissolved oxygen (DO) was kept at saturation using air stones in each culture tank via a single airline linked to a regenerative blower (Model R4P115, Gast Manufacturing, Benton Harbor, Michigan, USA). DO, salinity, and water temperature were measured twice daily using a YSI-2030 Pro digital oxygen/temperature meter (YSI Corporation, Yellow Springs, Ohio, USA), and total ammonia nitrogen (TAN) and nitrite nitrogen were measured twice per week using YSI 9300 photometer (YSI, Yellow Springs, Ohio, USA). The pH of the water was measured two times per week during the experimental period using a pHep (Hanna Instrument, Smithfield, Rhode Island, USA).

### 2.4. Body Composition Analysis

Ten fish from the initial stock population were sampled, and three fish euthanized with buffered MS-222 from each experimental tank were randomly collected at the end of the trial and stored at –20°C for body composition analysis. Prior to proximate analysis, whole fish were rigorously blended and chopped in a mixer according to methods described by the Association of Official Analytical Chemists [30]. Proximate composition and mineral contents of the whole pompano body were analyzed by Midwest Laboratories (Omaha, Nebraska, USA) using standard AOAC methods [31]. Gross energy content was determined using a semimicrobomb calorimeter (Model 6725, Parr Instrument Co. Moline, Illinois, USA) [32].

### 2.5. Hematocrit Analysis

To determine hematocrit value, wax-sealed capillary tubes were spun down in 5 min using a hematocrit IEC Clinical Centrifuge (International Equipment Co., Needham HTS., Massachusetts, USA) at the standard setting recommended for this procedure [33]. The hematocrit percentage results were then read using a micro-capillary reader (International Equipment Co., Needham HTS., Massachusetts, USA).

### 2.6. Serum Biochemistry Analysis

Blood samples were allowed to clot overnight at 4°C, followed by centrifugation at 15,000 × *g* for 5 min to collect serum [34]. Three serum samples from each tank were then pooled into one 100 μL composite sample. The serum biochemical parameters were analyzed using a VetScan VS2 analyzer (Abaxis Inc., Zoetis, Union City, California, USA).

### 2.7. Microbiome Analysis

Fecal samples from the distal intestine were collected from the same three fish per tank after bleeding across all three dietary treatments (basal, CFP-20, and FSC-2). The fish were dissected, and a 2 cm segment of the distal intestine, located ~1 cm from the vent internally, was carefully removed using ethanol-sterilized scalpels and forceps. The excised intestinal segment was placed in a sterile Petri dish. Using two sets of sterile forceps, the fecal material was gently pressed out of the distal intestine and transferred into a sterile 2 mL screw-cap tube (Fisher Scientific, Waltham, Massachusetts, USA). Scalpels were disinfected with a 10% bleach and 70% ethanol solution during the process. The collected samples were immediately flash-frozen in liquid nitrogen and stored at −80°C to maintain the integrity of the gut microbiome for subsequent analysis [35]. Microbiome analysis of fish digesta intestines, diet, and water samples was processed and analyzed with the ZymoBIOMICS Targeted Sequencing Service (Zymo Research, Irvine, California, USA). Metagenomic data obtained from Zymo Research were used for downstream analyses.

### 2.8. Statistical Analysis

Statistical analyses were performed with SAS 9.4 (SAS Institute, Cary, NC, USA), R 4.4.2 [36] and RStudio 2024.12.0.0467 [37], utilizing tidyverse [38], segmented [39] packages. For gut microbiota analysis, filtered and trimmed reads were processed using phyloseq [40] and vegan [41] packages. The Shapiro–Wilk test and Bartlett's test were used to check the residuals of all parametric parameters for normality and equal variances, respectively [42]. All data were analyzed using a one-way ANOVA analysis. Significant outcomes were tested post hoc using Tukey's Honest Significant Difference for multiple comparisons. Linear, quadratic, and broken-line regression models were evaluated for all parameters using the coefficient of determination (*R*^2^), Akaike information criterion correct (AICc), weighted AICc, relative likelihood, and evidence ratio [43]. Unless otherwise noted, one-way ANOVA was presented together with the *R*^2^ and *p*-value of the best-fit models. The relationship between CFP inclusion levels and performance parameters such as FW, WG, ANPR, and ER was plotted with 95% confidence intervals.

Within-sample diversity was assessed using richness, evenness, Shannon, and Invsimpson for α-diversity through one-way ANOVA. Read counts were normalized with metagenomeSeq v1.32.0 [44] and analyzed via principal-coordinate analysis (PCoA) based on Bray–Curtis and unweighted UniFrac distances for β-diversity. Permutational multivariate analysis of variance (PERMANOVA) with the adonis2 function determined differences in bacterial community centroids across diets. A priori value of a significance level of *α* = 0.05 was used for all statistical analyses.

## 3. Results

### 3.1. Water Quality

The water quality parameters assessed during the growth trial were maintained within acceptable values for the cultivation of Florida pompano at 6.77 ± 0.37 mg L^−1^ (DO), 27.51 ± 0.78°C (temperature), 11.76 ± 0.57 g L^−1^ (salinity), 8.02 ± 0.15 (pH), 0.27 ± 0.32 mg L^−1^ (TAN), and 0.26 ± 0.23 mg L^−1^ (nitrite), as presented in Table 3.

### 3.2. Growth Performance

**Figure 1:**
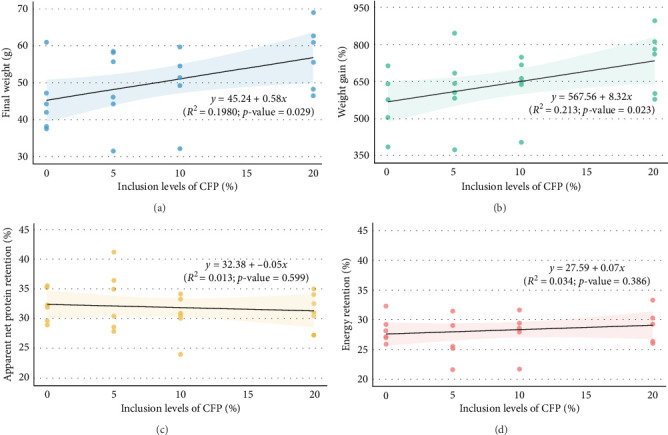
Relationship between various levels of corn fermented protein (CFP) and growth performance, including final weight (FW) (A), percentage weight gain (WG) (B), apparent net protein retention (ANPR) (C), and energy retention (D) of Florida pompano (*Trachinotus carolinus*) fed experimental diets during 12 weeks (*n* = 6).

During the growth trial, all experimental fish fully ingested the test feed, exhibiting no abnormal behavior during the trial. The growth performance data are presented in Table 4. No significant differences in growth performance of Florida pompano fed diets with different levels of CFP and FSC were found (*p*  > 0.05). Mean final fish weight ranged from 44.61 to 56.98 g, WG ranged between 557% and 738%, TGC varied from 0.55 to 0.72, FCR ranged from 1.56 to 1.75, and survival ranged from 82% to 92%. Mortalities occurring during the trials were unrelated to dietary treatment. There were no significant differences in terms of ANPR (*p* = 0.560), ER (*p* = 0.217), and HSI (*p* = 0.420) among treatments. Fish fed the CFP-20 diet showed slightly better growth performance values than other diets, with numerically higher FW and WG, although the data were not statistically significant. Linear regression analysis revealed significant relationships (*p*  < 0.05) between CFP inclusion levels and several growth metrics, including FW (*R*^2^ = 0.198, *p* = 0.029) (Figure 1A) and WG (*R*^2^ = 0.213, *p* = 0.023) (Figure 1B). In contrast, inclusion levels of CFP explained only 1.3% and 3.4% of the variation in ANPR (*p* = 0.599) and ER (*p* = 0.386), respectively (Figure 1C,D).

### 3.3. Proximate and Mineral in Whole-Body Fish

The whole-body proximate and mineral composition of fish was shown in Table 5. No statistically significant differences were observed among treatments for crude fat, energy, or ash content (*p*  > 0.05). Statistically significant differences were observed in dry matter (*p* = 0.019) and crude protein content (*p* = 0.0005) across treatments. Fish fed CFP-20 showed the highest dry matter content (32.56%), while crude protein content was significantly lower in CFP-10 and CFP-20 diets (53.96% and 53.06%, respectively) compared to other diets.

Regarding mineral analysis, magnesium (Mg) was significantly higher (*p* = 0.036) in fish fed with basal diet (0.14%) compared to the FSC-2 diet (0.11%), while no significant differences were observed in other mineral content across all dietary treatments. Regression analysis indicated that CFP inclusion explained ~20.7% of the variation in crude protein and 15.4% in crude lipid. For trace minerals, manganese and sodium concentrations were significantly influenced by CFP inclusion, exhibiting quadratic responses (*p*  < 0.05). Additionally, the quadratic regression model accounted for 13.1% and 21.9% of the variation in ash and zinc content, respectively, although these effects were not statistically significant (*p* = 0.229 and *p* = 0.075, respectively).

### 3.4. Blood Biochemistry

Table 6 presents the enzyme activities and serum levels of Florida pompano across various dietary treatments of CFP and FSC. No significant differences were observed between treatments in blood biochemistry and hematological parameters (*p*  > 0.05). Glucose levels were slightly lower in the CFP-5 diet (143.83 mg dL^−1^), and ALP activity was reduced in the CFP-20 diet (169.33 mg dL^−1^) compared to other diets; however, these variations were not statistically significant (*p* = 0.732), suggesting no adverse impact of the experimental diets on health indicators. Linear regression models demonstrated similar outcomes, with coefficients of determination ranging from less than 4% across hemolymph parameters and *p*-values greater than 0.05. Among all hemolymph parameters evaluated, hematocrit levels were the only variable that exhibited a quadratic response to CFP inclusion, though this effect was marginal (*R*^2^ = 0.248, *p* = 0.05).

### 3.5. Microbiome Diversity

The V3-V4 region of the 16S rRNA gene was subjected to high-throughput sequencing, which yielded 8,791,853 sequences that were distributed among 6324 amplicon sequence variants (ASVs). Three samples with less than 1000 reads, which belong to each treatment group (basal, CFP-20, and FSC-2), were removed to improve normalization and quality control, resulting in 31 samples (nine fecal samples per treatment, three diet samples, and one water sample) with a median read depth of 290,880 reads per sample. Rarefaction analysis curves indicated that much of the diversity was adequately captured and reached saturation for downstream analysis (Supporting Information 2: Figure S1).

**Figure 2:**
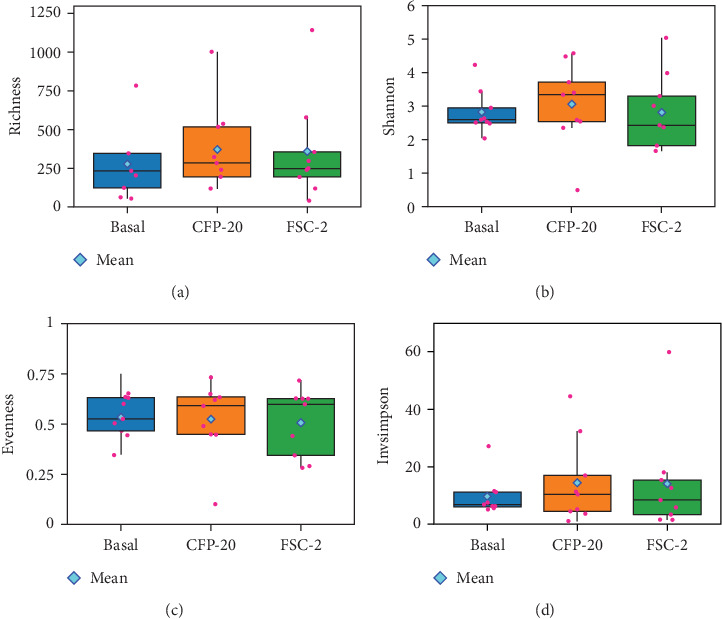
Richness (A), Shannon (B), evenness (C), and Invsimpson (D) boxplots of the α-diversity of Florida pompano fecal microbiome communities fed experimental diets for 12 weeks. CFP-20, corn fermented protein with 20% supplemented diet and FSC-2, *Saccharomyces cerevisiae* fermentation product 2% supplemented diet.

**Figure 3:**
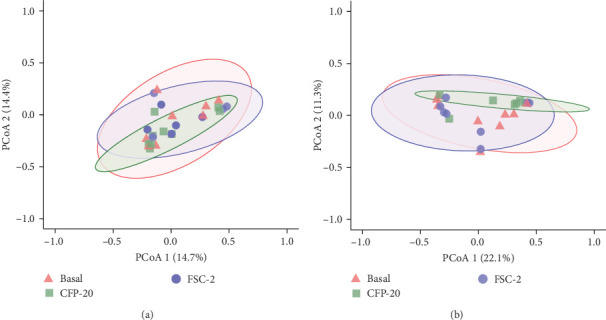
Principal coordinate analysis (PCoA) plot illustrates the β-diversity of microbial communities in Florida pompano fecal samples fed for 12 weeks with experimental diets based on Bray–Curtis dissimilarity (A) and unweighted UniFrac distances (B). Different shapes and colors represent experimental groups. CFP-20, corn fermented protein with 20% supplemented diet and FSC-2, *Saccharomyces cerevisiae* fermentation product 2% supplemented diet.

The intestinal microbiota results did not present significant differences for α and β-diversity metrics (Table 7). Bacteria richness, Shannon diversity, evenness, and Invsimpson metrics were used to estimate the α-diversity of each sample detected (one-way ANOVA, Figure 2A–D). There were no statistically significant differences in gut community richness between treatments (*p* = 0.313). Evenness was also similar across experimental groups (*p* = 0.606). Additionally, comparisons using the Shannon diversity index (*p* = 0.806) and inverse Simpson index (*p* = 0.778) indicated no significant treatment-related effects on community diversity. The β-diversity of the treatments was assessed through PCoA on normalized reads utilizing Bray–Curtis and unweighted UniFrac distances to determine the impact of various diets on the gut bacterial community composition of pompano following a 12-week culture period. The analysis is based on the permutation test for comparing multivariate differences in centroids, known as PERMANOVA. The Bray–Curtis distances of bacterial communities were similar across different dietary regimens (*p* = 0.381, *R*^2^ = 0.079, and *F* = 1.038). Besides, PCoA showed a sample separation based on the 14.7% and 14.4% variation explained by PCoA 1 and 2, respectively (Figure 3A). Concerning the unweighted UniFrac distance, which compares samples based on the proportion of unique species (Figure 3B), no significant treatment effect was observed (*p* = 0.372, *R*^2^ = 0.078, and *F* = 1.023).

**Figure 4:**
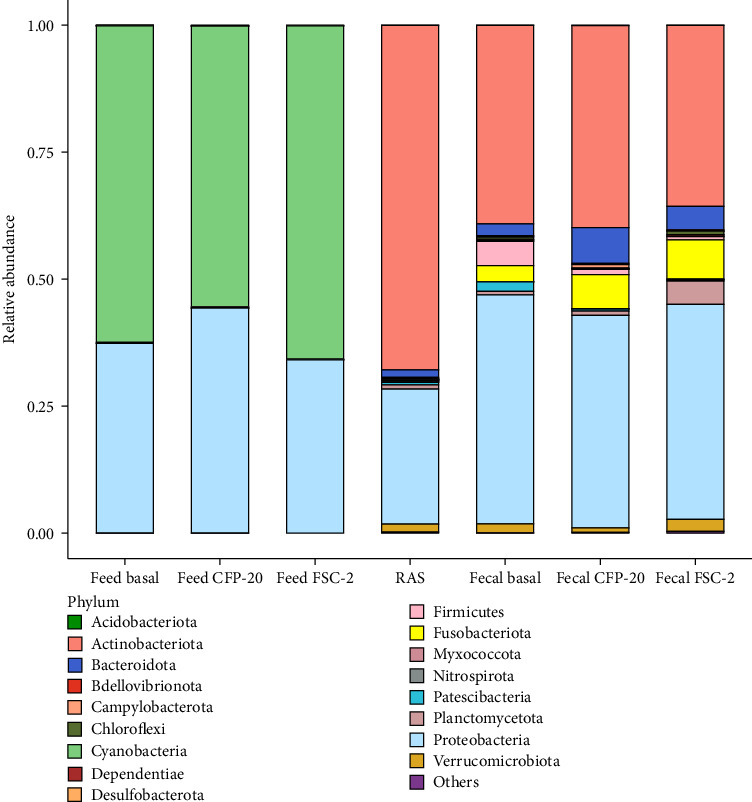
The abundance of composition at phylum level from feed, water (RAS), and fecal samples of Florida pompano (*Trachinotus carolinus*) fed experimental diets during 12 weeks. CFP-20, corn fermented protein with 20% supplemented diet and FSC-2, *Saccharomyces cerevisiae* fermentation product 2% supplemented diet.

**Figure 5:**
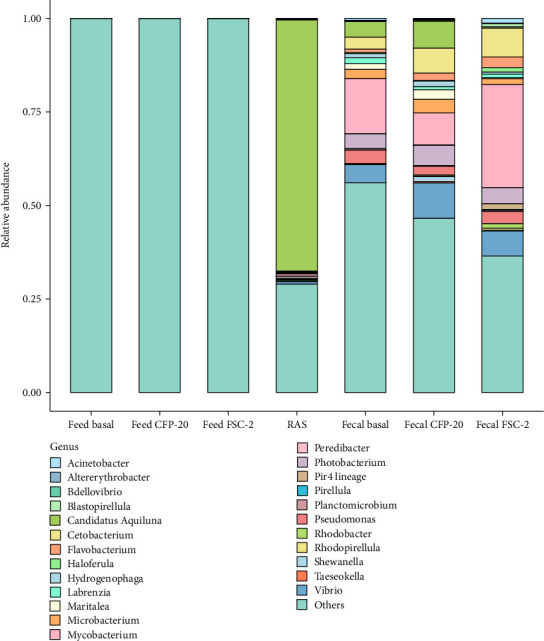
The abundance of composition at genus level from feed, water (RAS), and fecal samples of Florida pompano (*Trachinotus carolinus*) fed experimental diets during 12 weeks. CFP-20, corn fermented protein with 20% supplemented diet and FSC-2, *Saccharomyces cerevisiae* fermentation product 2% supplemented diet.

The overall composition of bacterial communities in fecal feed and water samples from Florida pompano based on treatment groups was illustrated at both the phylum and genus levels in Figures 4 and 5, respectively. *Proteobacteria* (43.09%) and *Actinobacteriota* (38.09%), *Fusobacteriota* (5.87%), *Bacteroidota* (4.59%), *and Firmicutes* (2.17%) were the dominant phyla in the fish fecal samples across all treatments, accounting for 93.80% of the total flora. At the genus level of fecal samples, *Mycobacterium* was the most abundant genus in the FSC-2 treatment (27.55%), followed by lower proportions in basal (14.74%) and CFP-20 (8.56%). Besides, the microbial composition of the feed samples from both diet treatments was notably similar, with *Cyanobacteria* (61.22%) and *Proteobacteria* (38.43%) identified as the predominant phyla. In contrast, the analysis of the water samples indicated that *Actinobacteriota* (67.79%) and *Proteobacteria* (26.54%) were the dominant phyla present. At the genus level of the feed samples, the microbial diversity of the treatment diets was dominated by unclassified taxa, with over 95% of sequences classified as “others.” Among the minor genera, *Pseudomonas* consistently appears in small proportions (0.04% to 0.06%), alongside trace contributions from *Acinetobacter*, *Flavobacterium*, *Microbacterium*, and *Mycobacterium*. In contrast, the water sample exhibited greater microbial diversity, with *Candidatus Aquiluna* accounting for the majority (67.08%).

## 4. Discussion

In this study, Florida pompano acclimated well to the culture system, accepting experimental diets with no evident palatability concerns with suitable water quality parameters [45, 46]. The growth performance of pompano fed diets CFP was comparable to that of fish fed basal and FSC diets over the 12-week trial. These results suggest that varying levels of CFP inclusion did not significantly affect the overall growth of Florida pompano under experimental conditions. Although no statistically significant differences were observed in protein retention (*p* = 0.560), ER (*p* = 0.217), and HSI (*p* = 0.420) among treatments, regression analysis revealed significant relationships (*p*  < 0.05) between CFP inclusion levels and key growth parameters with the models explaining 18.6%–21.3% of the variation. These findings suggest that CFP contributed to growth trends, with levels up to 20% showing potential benefits for performance and feed efficiency, indicating that higher inclusion rates may further optimize results. While HSI remained unaffected, it is a key biomarker for metabolic and physiological responses in fish, reflecting the liver's role in nutrient metabolism [47, 48]. The results of this study indicate that CFP can be effectively incorporated into pompano diets without adverse effects on growth and nutrient retention while also showing potential for enhancing performance at higher inclusion levels.

The findings from the linear regression analysis align with studies that report varying results regarding the impact of ethanol yeast-based coproducts on fish growth. Gause and Trushenski [49] argued that ethanol yeast, a coproduct of ethanol production, can be used up to 41.3% addition in the diet of sunshine bass (*Morone chrysops* × *Morone saxatilis*) without impairing the growth performance, though complete replacement resulted in decreased growth and higher FCR, likely due to palatability issues when FM was removed. Goda et al. [50] reported that juvenile European seabass (*Dicentrarchus labrax*) tolerated up to 18.75% inclusion of high-protein DDGS with phytase supplement, achieving improved growth performance, while Burns and Gatlin III [51] found that Nile tilapia (*Oreochromis niloticus*) exhibited optimal growth with up to 24.45% high-protein DDGS inclusion. However, previous studies reported that dietary inclusion of CFP at 20% for Atlantic salmon (*Salmo salar*) or 32.25% for channel catfish (*Ictalurus punctatus*) impaired growth performance [13, 52].

Growth response may be influenced by the processing technologies used to produce ethanol yeast-based coproducts. Fluid Quip's MSC system, used in this study, applies postfermentation mechanical separation to increase protein content by removing solubles without additives. Other methods, such as ICM Advanced Processing Package (prefermentation separation), ProCap (flocculant-based), and ST Equipment (electrostatic), alter protein composition differently. This study suggests that incorporating CFP up to 20% does not result in significant negative effects; however, growth outcomes may differ depending on species and diet formulation.

The whole-body composition presented the impacts of dietary CFP and FSC on nutritional deposition in Florida pompano. Despite the formulation of diets to be isonitrogenous and isolipidic (40% crude protein and 8% crude lipid), an increase in whole-body crude fat content was observed with higher CFP inclusion, especially in the CFP-20 group. This suggests that residual lipids in CFP, rich in polyunsaturated fatty acids such as linoleic acid, can promote lipid accumulation when dietary energy exceeds immediate metabolic requirements [53]. Additionally, the fermentation process used in CFP may increase lipid availability by breaking down complex structures within the feed matrix, thereby improving ER [54]. Notably, fish fed the CFP-10 and CFP-20 diets showed a significant decrease in crude protein content, which suggests a dilution effect or a metabolic shift toward lipid deposition at higher CFP inclusion levels. However, this reduction in protein whole-body content did not translate into reduced protein utilization efficiency, as there was no significant difference in ANPR across treatments (*p* = 0.560). These findings suggest that while a higher inclusion level of CFP may slightly change nutrient partitioning, it does not impact fish's ability to retain dietary protein.

The analysis of AAs in the experimental diets indicated slight differences in essential AAs (EAAs), particularly lysine, methionine, and arginine. Lysine levels declined from 2.33 g 100 g^−1^ in the basal diet to 2.01 g 100 g^−1^ in the CFP-20 diet, which may restrict protein synthesis and reduce whole-body protein content, though both values satisfy the species' established lysine requirement. Additionally, methionine and arginine showed minor reductions in higher CFP diets, which likely affected protein metabolism. Total AA levels remained consistent; however, minor variations in EAAs likely influenced protein retention and nutrient partitioning, resulting in a greater emphasis on lipid accumulation with increased CFP inclusion levels. Fermentation increases nutrient concentration, but may modify the structural matrix of the ingredient, affecting mineral utilization in fish [55]. In terms of mineral composition, the reduction in Mg content at a 20% CFP inclusion level suggests potential interactions between dietary components and mineral bioavailability. Similarly, the decrease in phosphorus levels with increasing CFP inclusion may reflect differences in phosphorus utilization or bone mineralization efficiency.

The hematological profile of cultivated fish can be used to evaluate and identify stress conditions and diseases that impact production performance, thereby assessing their health and physiological condition [56]. In this study, serum glucose levels ranged from 143.83 to 169.33 mg dL^−1^, which falls within the normal physiological range for marine teleosts, indicating the absence of metabolic stress or disturbances [57]. Moreover, hematocrit levels, ranging from 40.17% to 47.17%, aligned with recognized reference values for marine species, indicating a sufficient oxygen-carrying capacity and general health [58]. The stability of blood biochemistry across different diets in our study is highlighted by the absence of significant changes in other serum biochemical parameters, including liver function markers (albumin, alkaline phosphatase, and alanine aminotransferase), as well as mineral homeostasis (calcium, phosphorus, sodium, and potassium). These findings are consistent with previous studies on channel catfish (*Ictalurus punctatus*), gilthead seabream (*Sparus aurata*), rainbow trout (*Oncorhynchus mykiss*), and cobia (*Rachycentron canadum*), which indicated that diets containing corn-derived proteins did not negatively impact health or physiological parameters [12, 59–61]. The data from this study suggests that the inclusion of CFP and FSC is safe and does not disrupt health indicators in juvenile Florida pompano.

Gut microorganisms play a crucial role in nutrition, facilitating the effective absorption of essential nutrients and generating daily energy [62]. They support the host's health by enabling digestion and breakdown of complex food molecules, thereby enhancing overall gut function [63]. The composition and diversity of the gut microbiota are highly influenced by dietary inputs, making diet one of the most critical environmental factors shaping the microbial community [64]. In this study, we investigated the effects of dietary basal, CFP-20, and FSC-2 on the intestinal microbiota of Florida pompano (*Trachinotus carolinus*) throughout a feeding trial. The richness and diversity of gut microbial communities are strongly linked to fish health and growth [65]. In aquaculture, maintaining microbial homeostasis is particularly important, as imbalances or dysbiosis can lead to poor nutrient utilization, reduced growth performance, and increased susceptibility to diseases [66]. Microbial homeostasis refers to the balanced and stable state of the gut microbiota, where the composition and diversity of microbial communities remain within a functional equilibrium. This stability ensures that gut microbiota can perform its essential roles, such as aiding digestion, nutrient absorption, immune modulation, and pathogen resistance, without being disrupted by external factors, including diet or environmental stressors [67]. In this study, we found that there was no significant difference in α-diversity metrics (within-sample diversity) and β-diversity analysis (diversity among different samples) in gut bacterial community composition across treatments (*p*  > 0.05). The observed stability in gut microbial diversity among dietary treatments suggests that the diets containing CFP-20 and FSC-2 provided adequate nutritional support to preserve microbial homeostasis in Florida pompano. PCoA showed distinct clustering patterns among dietary treatments, suggesting that while overall microbial diversity remained stable, certain compositional shifts occurred. The CFP-20 group exhibited a more tightly clustered microbiome profile compared to the basal and FSC-2 groups, indicating greater microbial stability, whereas the FSC-2 treatment displayed higher variability, potentially reflecting individual differences in microbial adaptation to the diet.

This aligns with findings from previous research showing that nutritionally balanced diets tend to maintain microbial stability by meeting the metabolic needs of both the host and its associated microbiota [68, 69]. The adaptability of pompano's gut microbiota to varied dietary inputs can also be attributed to its inherent resilience and functional redundancy [70]. Resilient microbial communities can resist or recover from perturbations, while functional redundancy ensures that essential functions are preserved even when specific microbial taxa fluctuate [71]. Furthermore, ingredients such as *Saccharomyces cerevisiae* in the FSC-2 diet likely contributed to this stability by promoting beneficial bacterial taxa and supporting gut epithelial integrity, as highlighted in previous studies [72–74].

The phyla *Proteobacteria*, *Actinobacteria*, *Fusobacteriota*, *Bacteroidetes*, and *Firmicutes* were found in decreasing order of relative abundance in marine fish gut microbiota [75]. In this study, *Proteobacteria* were the dominant type of bacteria in the gut microbiota of pompano due to their metabolic versatility and role in nutrient cycling particularly in carbon, nitrogen, and sulfur [71, 76]. This dominance may be attributed to the composition of the CFP-20 and FSC-2 diets, both known to provide nutrients that support microbial growth. CFP is rich in fermentable carbohydrates, peptides, and micronutrients, which are substrates known to enhance the abundance of *Proteobacteria* and *Firmicutes* in the gut, as these phyla are involved in fermentative metabolism and short-chain fatty acid production [77, 78]. The high abundance of *Actinobacteriota* observed is noteworthy, as members of this phylum are known for their ability to produce antimicrobial compounds and modulate immune responses, potentially benefiting host health [79]. The CFP-20 and FSC-2 diets may also influence the prevalence of *Actinobacteriota* through their provision of functional proteins and nutrients that encourage microbial colonization. For example, CFP provides bioactive peptides that might interact with gut epithelial cells, promoting the growth of *Actinobacteriota*, which thrive in nutrient-rich environments [80]. Additionally, the fermentation process of CFP enhances the bioavailability of EAA and organic acids, which could further support microbial diversity and functionality [11, 81, 82].

At the genus level, *Mycobacterium* was the most abundant in the FSC-2 treatment group. While *Mycobacterium* is linked to immune modulation, some species are opportunistic pathogens, raising questions about potential trade-offs between immune benefits and infection risks, which future studies should address this balance [83]. The diet inclusion of FSC at 2% was intentional because its active ingredient, *Saccharomyces cerevisiae* yeast, is well known for promoting gut health in fish [84, 85]. This allowed for direct comparison to CFP-20, which contains a variable yeast fraction (~20%–25% of dry matter), though the total yeast content was not standardized across diets. The FSC-2 diet thus served as a functional benchmark to evaluate CFP's performance beyond its yeast component alone. Research has demonstrated that this yeast can modulate cytokine expression, enhance the integrity of the gut epithelium, and strengthen immune responses [24, 86]. Using FSC-2 as a baseline allowed us to evaluate CFP-20 diets in comparison to a proven gut health promoter. While direct studies on CFP's effects on gut microbiota are limited, its inclusion at 20% aimed to assess its potential as a sustainable protein source. The FSC diet also served to counterbalance risks, such as increased *Mycobacterium* abundance, offering a comparative framework to evaluate CFP's role in fish diet.

Regarding feed samples, the microbial composition was dominated by *Cyanobacteria* and *Proteobacteria*, reflecting the environmental origin of feed ingredients and production processes. Notably, over 95% of sequences were classified as “others,” highlighting the presence of uncharacterized microbial taxa. This aligns with findings from Karlsen et al. [87], who emphasized the significant, yet incompletely comprehended, function of unidentified taxa in fish diets. Further research to characterize these taxa may offer insights into their functional roles and interactions within the aquaculture environment. Water samples, in contrast, exhibited higher microbial diversity, with *Actinobacteria* and *Proteobacteria* as dominant phyla. The high abundance of *Candidatus Aquiluna*, a known contributor to nitrogen cycling, suggests its potential to improve water quality in aquaculture systems, though its specific effects on fish health remain unclear [88].

The observed stability in gut microbiota across dietary treatments underscores the adaptability of Florida pompano to dietary inputs and highlights CFP-20 and FSC-2 as viable components in feeds. Targeted nutritional strategies, including plant-based diets and functional additives, could enhance gut microbiota functionality and increase resilience to environmental stressors and disease [89, 90]. Future studies should focus on elucidating the mechanisms by which CFP interacts with gut microbiota and evaluating its long-term effects on fish health and productivity.

## 5. Conclusion

CFP, a newly developed ingredient from the ethanol industry, is produced using advanced processing technology that enhances its nutritional profile compared to traditional ethanol co-products. This study demonstrates that diets, including CFP and FSC, can serve as effective protein sources and/or feed additives in practical diets for Florida pompano. Notably, there were no significant differences among the three types of diets in terms of gut microbiota, blood biochemistry, and growth performance. These findings provide strong evidence that CFP is a sustainable alternative protein source, offering a novel solution to reduce dependence on traditional protein sources while expanding ingredient options for feed formulation and supporting the long-term sustainability of marine fish production.

## Figures and Tables

**Table 1:** Composition of diets (40% protein and 8% lipid on an as-is basis) contains various levels of corn fermented protein (CFP) and fermented *Saccharomyces cerevisiae* (FSC) fed to juvenile Florida pompano (*Trachinotus carolinus*) for 12 weeks.

Ingredients	Basal	CFP-5	CFP-10	CFP-20	FSC-2
Poultry by-product meal^a^	15.00	15.00	15.00	15.00	15.00
Soybean meal^b^	52.00	46.45	40.87	29.75	51.15
Corn protein concentrate^c^	6.75	6.75	6.75	6.75	6.75
Corn fermented protein^d^	0.00	5.00	10.00	20.00	0.00
Fermented yeast^e^	0.00	0.00	0.00	0.00	2.00
Fish oil^f^	3.50	3.50	3.50	3.50	3.50
Soy oil	1.68	1.46	1.23	0.79	1.66
Lecithin^g^	0.50	0.50	0.50	0.50	0.50
Corn starch^h^	7.27	8.04	8.85	10.41	6.14
Whole wheat^h^	10.00	10.00	10.00	10.00	10.00
Mineral premix^i^	0.25	0.25	0.25	0.25	0.25
Vitamin premix^j^	0.50	0.50	0.50	0.50	0.50
Choline chloride^h^	0.20	0.20	0.20	0.20	0.20
Stay-C^k^	0.10	0.10	0.10	0.10	0.10
CaP-dibasic^h^	1.75	1.75	1.75	1.75	1.75
Taurine^l^	0.50	0.50	0.50	0.50	0.50
Proximate composition (g 100 g^−1^, as is)^m^					
Moisture	7.81	7.75	7.50	7.40	7.38
Crude protein	41.99	41.61	41.82	41.91	41.68
Crude fat	8.43	8.32	8.45	8.46	8.77
Crude fiber	2.94	3.00	3.07	3.36	3.18
Ash	7.13	6.74	6.55	5.97	7.19

^a^Darling Ingredients Inc., Dallas, Texas, USA.

^b^Solvent Extracted Soybean Meal, Auburn University, Auburn, Alabama, USA.

^c^Empyreal 75 Cargill Corn Milling, Cargill Inc., Blair, Nebraska, USA.

^d^Altipro, Renewable Products Marketing group, Shakopee, Minnesota, USA.

^e^DVaqua, Diamond V, Cedar Rapids, Iowa, USA.

^f^Omega Protein Inc., Reedville, Virginia, USA.

^g^The Solae Company, St. Louis, Missouri, USA.

^h^MP Biochemicals Inc., Solon, Ohio, USA.

^i^Mineral premix (g 100 g^−1^ premix): cobalt chloride, 0.008; cupric sulfate pentahydrate, 0.01; ferrous sulfate heptahydrate, 20; manganous sulfate anhydrous, 2.90; potassium iodide, 0.240; sodium selenite, 0.048; zinc sulfate heptahydrate, 17.600; and α cellulose 59.194.

^j^Vitamin premix (g kg^−1^ premix): thiamin HCL, 8.0; riboflavin, 8.0; pyridoxine HCl, 5.0; Ca-pantothenate, 20.0; niacin, 40.0; biotin, 0.040; folic acid, 1.80; cyanocobalamin, 0.002; vitamin A acetate (500,000 IU g^−1^), 2.40; vitamin D₃ (400,000 IU g^−1^); 0.50; DL-α-tocopheryl acetate, 80.0; and α cellulose, 834.258.

^k^Stay C, (L-ascorbyl-2-polyphosphate 35% Active C), Roche Vitamins Inc., Parsippany, New Jersey, USA.

^l^Aldrich-Sigma, St. Louis, Missouri, USA.

^m^Analysis conducted by University of Missouri Agricultural Experimental Station Chemical Laboratories (Columbia, Missouri, USA).

**Table 2:** Amino acid analysis (g 100 g^−1^, as-is) of the experimental diets fed to Florida pompano (*Trachinotus carolinus*) for 12 weeks.

Amino acids	Basal	CFP-5	CFP-10	CFP-20	FSC-2
Essential amino acids (EAAs)					
Arginine	2.63	2.56	2.52	2.29	2.66
Histidine	1.04	1.04	1.04	1.02	1.04
Isoleucine	1.90	1.91	1.90	1.84	1.89
Leucine	3.58	3.77	3.85	4.18	3.64
Lysine	2.33	2.27	2.24	2.01	2.34
Methionine	0.70	0.73	0.75	0.75	0.72
Phenylalanine	2.04	2.08	2.09	2.11	2.07
Threonine	1.50	1.52	1.53	1.51	1.55
Tryptophan	0.48	0.44	0.43	0.41	0.47
Valine	2.08	2.09	2.10	2.07	2.04
Nonessential amino acids (NEAAs)					
Alanine	2.23	2.32	2.36	2.54	2.26
Aspartic acid	3.94	3.87	3.76	3.47	3.98
Cysteine	0.63	0.65	0.65	0.65	0.63
Glutamic acid	7.39	7.42	7.34	7.30	7.44
Glycine	2.11	2.07	2.09	2.02	2.13
Hydroxylysine	0.03	0.03	0.04	0.04	0.04
Hydroxyproline	0.46	0.42	0.33	0.29	0.32
Lanthionine	0.14	0.15	0.16	0.14	0.15
Ornithine	0.08	0.07	0.07	0.07	0.07
Proline	2.37	2.50	2.56	2.75	2.42
Serine	1.62	1.63	1.65	1.66	1.70
Taurine	0.75	0.78	0.77	0.75	0.75
Tyrosine	1.51	1.54	1.54	1.56	1.54
Total amino acids	41.54	41.86	41.77	41.43	41.85

*Note:* analysis conducted by University of Missouri Agricultural Experimental Station Chemical Laboratories (Columbia, Missouri, USA).

Abbreviations: CFP, corn fermented protein; FSC, fermented *Saccharomyces cerevisiae*.

**Table 3:** The water quality parameters for Florida pompano (*Trachinotus carolinus*) were reared in a growth trial in a clear water recirculating system for 12 weeks.

Parameters	Mean ± SD
Dissolved oxygen (mg L^−1^)	6.77 ± 0.37
Temperature (°C)	27.51 ± 0.78
Salinity (g L^−1^)	11.76 ± 0.57
pH	8.02 ± 0.15
Total ammonia nitrogen (mg L^−1^)	0.27 ± 0.32
Nitrite nitrogen (mg L^−1^)	0.26 ± 0.23

*Note:* values are shown as mean ± standard deviation.

**Table 4:** Growth performance of Florida pompano (*Trachinotus carolinus*) with an initial weight of 6.08 ± 0.55 g (mean ± standard deviation) cultured in a clear water recirculating system during 12 weeks on experimental diets (40% protein and 8% lipid on an as-is basis).

Diets	Final weight (g)	Weight gain (%)	TGC	FCR	Survival (%)	ANPR (%)	ER (%)	HSI (%)
Basal	45.00	565	0.55	1.70	83.33	32.22	28.27	0.95
CFP-5	48.99	622	0.61	1.64	81.67	33.22	26.95	1.10
CFP-10	50.17	636	0.63	1.64	81.67	30.48	28.48	1.05
CFP-20	56.98	738	0.72	1.56	91.67	31.70	29.24	1.10
FSC-2	44.61	557	0.55	1.75	88.33	30.30	25.41	1.01
One-way ANOVA								
PSE	3.53	48	0.05	6.25	6.25	1.40	1.21	0.05
*p*-Value	0.123	0.085	0.110	0.311	0.728	0.560	0.217	0.420
Regression*⁣*^*∗*^								
*R*^2^	0.198	0.213	0.208	0.091	0.053	0.013	0.034	0.186
*p*-Value	0.029	0.023	0.025	0.152	0.281	0.599	0.386	0.035

*Note:* Values represent the mean of six replicates of each diet.

Abbreviations: ANPR, apparent net protein retention; CFP, corn fermented protein; ER, energy retention; FCR, feed conversion ratio; FSC, fermented *Saccharomyces cerevisiae*; HSI, hepatosomatic index; PSE, pooled standard error; TGC, thermal growth coefficient.

*⁣* ^*∗*^Linear regression based on basal and different concentrations of CFP in diets.

**Table 5:** Proximate and mineral analysis of whole-body (g 100 g^−1^ dry weight) of Florida pompano (*Trachinotus carolinus*) from the growth trial fed graded levels of experimental diets (40% protein and 8% lipid on an as-is basis) cultured in a clear water recirculating system for 12 weeks.

Proximate analysis	Basal	CFP-5	CFP-10	CFP-20	FSC-2	One-way ANOVA		Regression*⁣*^*∗*^	
						PSE	*p*-Value	*R* ^2^	*p*-Value
Dry matter (%)*⁣*^*∗∗*^	30.16^a,b^	29.42^b^	31.60^a,b^	32.56^a^	29.88^a,b^	0.676	0.019	0.063	0.226
Crude protein (%)*⁣*^*∗∗*^	60.65^a^	60.58^a^	53.96^b^	53.06^b^	60.32^a^	1.37	0.0005	0.207	0.078
Crude fat (%)	28.15	26.00	28.97	31.70	29.53	1.71	0.241	0.154	0.058
Energy (cal g^−1^)	5801	5802	5802	5854	5865	47.00	0.797	0.057	0.204
Ash (%)	12.67	9.60	11.05	11.89	10.44	0.93	0.189	0.131	0.229
Sulfur (%)	0.90	0.90	0.83	0.84	0.86	0.03	0.295	0.156	0.056
Phosphorus (%)	2.50	2.24	2.19	1.97	1.86	0.19	0.200	0.132	0.081
Potassium (%)	1.13	1.14	1.06	1.06	1.08	0.04	0.427	0.125	0.090
Magnesium (%)	0.14^a^	0.13^a,b^	0.13^a,b^	0.11^a,b^	0.11^b^	0.01	0.036	0.217	0.022
Calcium (%)	3.79	3.30	3.24	2.82	2.58	0.38	0.226	0.116	0.104
Sodium (%)	0.41	0.39	0.35	0.35	0.36	0.02	0.038	0.367	0.008
Iron (ppm)	38.18	41.02	38.98	42.67	36.73	2.66	0.550	0.042	0.338
Manganese (ppm)	10.27	7.22	7.07	6.95	7.10	0.92	0.077	0.273	0.035
Zinc (ppm)	73.83	65.92	58.20	63.88	56.57	4.24	0.059	0.219	0.075

*Note:* values represent the mean of six replicates of each diet. Means not sharing any letter are significantly different by the Tukey's HSD-test at the 5% level of significance.

Abbreviations: CFP, corn fermented protein; FSC, fermented *Saccharomyces cerevisiae*; PSE, pooled standard error.

*⁣* ^*∗*^Linear and quadratic regressions based on basal and different concentrations of CFP in diets.

*⁣* ^*∗∗*^Five replicates for CFP-10 and CFP-20 and six replicates for other treatments.

**Table 6:** Blood biochemistry parameters of Florida pompano (*Trachinotus carolinus*) cultured in the recirculating system for 12 weeks, fed different experimental diets (40% protein and 8% lipid on an as-is basis).

Parameters	Basal	CFP-5	CFP-10	CFP-20	FSC-2	One-way ANOVA		Regression*⁣*^*∗*^	
						PSE	*p*-Value	*R* ^2^	*p*-Value
Albumin (U L^−1^)	2.75	2.82	3.03	3.00	2.73	0.28	0.903	0.020	0.506
Alkaline phosphatase (U L^−1^)	8.83	10.00	9.00	5.00	7.17	4.35	0.934	0.028	0.432
Alanine aminotransferase (U L^−1^)	1.50	1.41	1.31	1.50	1.36	0.15	0.795	0.000	0.948
Amylase (U L^−1^)	14.83	18.83	11.83	13.17	27.83	6.77	0.474	0.006	0.713
Total bilirubin (mg dL^−1^)	0.22	0.22	0.22	0.23	0.28	0.05	0.801	0.003	0.792
Urea nitrogen (mg dL^−1^)	0.33	0.50	0.67	0.33	0.50	0.22	0.795	0.000	0.948
Calcium (mg dL^−1^)	14.98	14.90	15.17	14.80	15.47	0.35	0.701	0.003	0.787
Phosphorus (mg dL^−1^)	15.20	15.03	16.05	15.28	15.55	0.77	0.897	0.002	0.827
Creatinine (mg dL^−1^)	0.02	0.03	0.02	0.00	0.00	0.02	0.678	0.032	0.402
Glucose (mg dL^−1^)	169.00	143.83	156.33	156.67	169.33	14.90	0.732	0.003	0.799
Sodium (mmol L^−1^)	172.50	171.00	169.00	170.17	173.17	2.05	0.609	0.029	0.426
Potassium (mmol L^−1^)	8.07	8.10	8.35	8.57	7.57	0.98	0.962	0.008	0.679
Total protein (g dL^−1^)	4.57	4.78	4.83	4.62	4.78	0.17	0.763	0.000	0.976
Globulin (g dL^−1^)	1.83	1.75	1.55	1.47	2.05	0.26	0.548	0.045	0.320
Hematocrit (%)	40.83	40.94	43.53	40.17	47.17	2.42	0.252	0.248	0.050

*Note:* values represent the mean of six replicates of each diet. Means not sharing any letter are significantly different by the Tukey's HSD-test at the 5% level of significance.

Abbreviations: CFP, corn fermented protein; FSC, fermented *Saccharomyces cerevisiae*.

*⁣* ^*∗*^Linear regression based on basal and different concentrations of CFP in diets.

**Table 7:** Results of statistical analysis of α and β-diversity of Florida pompano (*Trachinotus carolinus*) intestinal microbiota fed different experimental diets after 12 weeks.

α-Diversity		β-Diversity		
	*p*-Value		*R* ^2^	*p*-Value
Observed features	0.313	Bray–Curtis	0.0796	0.381
Evenness	0.606	Unweighted UniFrac	0.0785	0.372
Shannon	0.806	Weighted UniFrac	0.055	0.822
Inverse Simpson	0.778	—	—	—

## Data Availability

Data are available upon request from the authors. Raw sequences for microbiome analysis have been deposited in the NCBI GenBank BioProject (PRJNA1222464). The R codes for reproducing our microbiome results are available at the GitHub repository (https://github.com/Nhvtrih/Pompano_CFP).

## Supporting Information

Supporting Information 1 The arrival checklist has been attached as a supporting document with all experiment details reported.

Supporting Information 2 Figure S1. Rarefaction curve sequences showing the microbial community complexities and reads per sample following normalization. CFP-20, corn fermented protein with 20% supplemented diet; FSC-2, Saccharomyces cerevisiae fermentation product 2% supplemented diet; and OTUs, operational taxonomic units.
