# Supplementary material for: Growth Performance, Blood Chemistry, and Intestinal Bacterial Community of Florida Pompano (Trachinotus carolinus) Fed Different Levels of Corn Fermented Protein and Yeast Diets
Source: Aquac Nutr. 2025 Aug 22;2025:8872997. doi: 10.1155/anu/8872997 (PMC12396910; doi:10.1155/anu/8872997)
Supplement: Supporting Information 1 — The arrival checklist has been attached as a supporting document with all experiment details reported. [file 8872997.f1.docx]

**Reporting checklist for study using laboratory animals**

The manuscript “Growth performance, blood chemistry and intestinal bacterial community of Florida Pompano (*Trachinotus carolinus*) fed different levels of corn fermented protein and yeast diets” with ID No. 8872997. A completed arrive checklist has been completed as required with the following table.

| **Essential 10/** Items |  | Reporting Item | Section/Lines number |
| --- | --- | --- | --- |
| Study design | [#1a](https://www.goodreports.org/reporting-checklists/arrive2/info/#1a) | Give details of the groups being compared, including control groups. If no control group has been used, the rationale should be stated. | Section 2.1: Experimental diets. Lines: 105-109 |
|  | [#1b](https://www.goodreports.org/reporting-checklists/arrive2/info/#1b) | Give details of the experimental unit (e.g., a single animal, litter, or cage of animals). | Section 2.2: Growth trial. Lines: 135-139 |
| Sample size | [#2a](https://www.goodreports.org/reporting-checklists/arrive2/info/#2a) | Specify the exact number of experimental units allocated to each group, and the total number in each experiment. Also indicate the total number of animals used. | Section 2.2: Growth trial. Lines: 135-139 |
|  | [#2b](https://www.goodreports.org/reporting-checklists/arrive2/info/#2b) | Explain how the sample size was decided. Provide details of any a priori sample size calculation, if done. | Section 2.2: Growth trial. Lines: 137-139 |
| Inclusion and exclusion criteria | [#3a](https://www.goodreports.org/reporting-checklists/arrive2/info/#3a) | Describe any criteria used for including or excluding animals (or experimental units) during the experiment, and data points during the analysis. Specify if these criteria were established a priori. If no criteria were set, state this explicitly. | Section 2: Materials and Methods  Lines: 188-190 |
|  | [#3b](https://www.goodreports.org/reporting-checklists/arrive2/info/#3b) | For each experimental group, report any animals, experimental units, or data points not included in the analysis and explain why. If there were no exclusions, state so. | No exclusion |
|  | [#3c](https://www.goodreports.org/reporting-checklists/arrive2/info/#3c) | For each analysis, report the exact value of n in each experimental group. | Section 2: Materials and Methods.  Lines: 135-137  Lines: 152-153  Lines: 188-190  Lines: 206-207  Lines: 211-212  Lines: 925-926  Lines: 935  Lines: 939  Lines: 951 |
| Randomisation | [#4a](https://www.goodreports.org/reporting-checklists/arrive2/info/#4a) | State whether randomisation was used to allocate experimental units to control and treatment groups. If done, provide the method used to generate the randomisation sequence. | Section 2: Materials and Methods. Lines 135-137 |
|  | [#4b](https://www.goodreports.org/reporting-checklists/arrive2/info/#4b) | Describe the strategy used to minimise potential confounders such as the order of treatments and measurements, or animal/cage location. If confounders were not controlled, state this explicitly. | Section 2: Materials and Methods.  Lines: 130-133  Lines: 135-139  Lines: 142-148 |
| Blinding | [#5](https://www.goodreports.org/reporting-checklists/arrive2/info/#5) | Describe who was aware of the group allocation at the different stages of the experiment (during the allocation, the conduct of the experiment, the outcome assessment, and the data analysis). | The study did not use blinding during allocation, outcome assessment, or analysis. However, data were analyzed using coded treatment labels to reduce bias. |
| Outcome measures | [#6a](https://www.goodreports.org/reporting-checklists/arrive2/info/#6a) | Clearly define all outcome measures assessed (e.g., cell death, molecular markers, or behavioural changes). | Section 2: Materials and Methodology. Lines: 144-168. |
|  | [#6b](https://www.goodreports.org/reporting-checklists/arrive2/info/#6b) | For hypothesis-testing studies, specify the primary outcome measure, i.e., the outcome measure that was used to determine the sample size. | Section 1: Introduction. Lines: 98-101 |
| Statistical methods | [#7a](https://www.goodreports.org/reporting-checklists/arrive2/info/#7a) | Provide details of the statistical methods used for each analysis, including software used. | Section 2.8: Statistical analysis. Lines 226-245 |
|  | [#7b](https://www.goodreports.org/reporting-checklists/arrive2/info/#7b) | Describe any methods used to assess whether the data met the assumptions of the statistical approach, and what was done if the assumptions were not met. | Section 2.8: Statistical analysis. Lines 226-245 |
| Experimental animals | [#8a](https://www.goodreports.org/reporting-checklists/arrive2/info/#8a) | Provide species-appropriate details of the animals used, including species, strain and substrain, sex, age or developmental stage, and, if relevant, weight. | Section 2.2: Growth trial. Lines 122-126 |
|  | [#8b](https://www.goodreports.org/reporting-checklists/arrive2/info/#8b) | Provide further relevant information on the provenance of animals, health/immune status, genetic modification status, genotype, and any previous procedures. | Section 2.2: Growth trial. Lines: 122-126 |
| Experimental procedures | [#9a](https://www.goodreports.org/reporting-checklists/arrive2/info/#9a) | For each experimental group, including controls, describe the procedures in enough detail to allow others to replicate what was done, how it was done, and what was used. | Section 2: Materials and methods.  Lines: 103-218 |
|  | [#9b](https://www.goodreports.org/reporting-checklists/arrive2/info/#9b) | Timing and frequency of procedures | Lines: 142-145 |
|  | [#9c](https://www.goodreports.org/reporting-checklists/arrive2/info/#9c) | Where procedures were carried out (including detail of any acclimatisation periods). | Lines: 126-130 |
|  | [#9d](https://www.goodreports.org/reporting-checklists/arrive2/info/#9d) | Rationale for procedures | Lines: 145-148  Lines: 153-161 |
| Results | [#10a](https://www.goodreports.org/reporting-checklists/arrive2/info/#10a) | For each experiment conducted, including independent replications, report summary/descriptive statistics for each experimental group, with a measure of variability where applicable (e.g., mean and SD, or median and range). | Section 3: Results  Lines: 248-341 |
|  | [#10b](https://www.goodreports.org/reporting-checklists/arrive2/info/#10b) | If applicable, for each experiment conducted, including independent replications, report the effect size with a confidence interval. | Effect size and confidence interval are not reported. Regression R², PSE and p-values used instead. |
| **Recommended set** |  |  |  |
| Abstract | [#11](https://www.goodreports.org/reporting-checklists/arrive2/info/#11) | Provide an accurate summary of the research objectives, animal species, strain and sex, key methods, principal findings, and study conclusions. | Abstract  Lines: 19-38 |
| Background | [#12a](https://www.goodreports.org/reporting-checklists/arrive2/info/#12a) | Include sufficient scientific background to understand the rationale and context for the study, and explain the experimental approach. | Section 1: Introduction. Lines: 40-101 |
|  | [#12b](https://www.goodreports.org/reporting-checklists/arrive2/info/#12b) | Explain how the animal species and model used address the scientific objectives and, where appropriate, the relevance to human biology. | Section 1: Introduction. Lines: 40-101 |
| Objectives | [#13](https://www.goodreports.org/reporting-checklists/arrive2/info/#13) | Clearly describe the research question, research objectives and, where appropriate, specific hypotheses being tested. | Section 1: Introduction. Lines: 98-101 |
| Ethical statement | [#14](https://www.goodreports.org/reporting-checklists/arrive2/info/#14) | Provide the name of the ethical review committee or equivalent that has approved the use of animals in this study and any relevant licence or protocol numbers (if applicable). If ethical approval was not sought or granted, provide a justification. | Ethical statement  Lines: 543-545 |
| Housing and husbandry | [#15](https://www.goodreports.org/reporting-checklists/arrive2/info/#15) | Provide details of housing and husbandry conditions, including any environmental enrichment. | Section 2.2: Growth trial, Lines: 127-129 |
| Animal care and monitoring | [#16a](https://www.goodreports.org/reporting-checklists/arrive2/info/#16a) | Describe any interventions or steps taken in the experimental protocols to reduce pain, suffering, and distress. | Section 2.2 Growth trial  Lines: 145-161 |
|  | [#16b](https://www.goodreports.org/reporting-checklists/arrive2/info/#16b) | Report any expected or unexpected adverse events. | Section 3.2: Growth performance. Lines: 260-261 |
|  | [#16c](https://www.goodreports.org/reporting-checklists/arrive2/info/#16c) | Describe the humane endpoints established for the study, the signs that were monitored, and the frequency of monitoring. If the study did not set humane endpoints, state this. | All procedures were conducted in accordance with the approved IACUC protocol. |
| Interpretation/scientific implications | [#17a](https://www.goodreports.org/reporting-checklists/arrive2/info/#17a) | Interpret the results, taking into account the study objectives and hypotheses, current theory, and other relevant studies in the literature. | Section 4: Discussion  Lines: 341-504 |
| Interpretation/scientific implications | [#17b](https://www.goodreports.org/reporting-checklists/arrive2/info/#17b) | Comment on the study limitations, including potential sources of bias, limitations of the animal model, and imprecision associated with the results. | Section 4: Discussion  Lines: 505-507 |
| Generalisability/translation | [#18](https://www.goodreports.org/reporting-checklists/arrive2/info/#18) | Comment on whether, and how, the findings of this study are likely to generalise to other species or experimental conditions, including any relevance to human biology (where appropriate). | Section 4: Discussion  Lines: 341-504 |
| Protocol registration | [#19](https://www.goodreports.org/reporting-checklists/arrive2/info/#19) | Provide a statement indicating whether a protocol (including the research question, key design features, and analysis plan) was prepared before the study, and if and where this protocol was registered. | A study protocol outlining the research question, design, and analysis plan was prepared internally before the experiment but was not registered in a public repository |
| Data access | [#20](https://www.goodreports.org/reporting-checklists/arrive2/info/#20) | Provide a statement describing if and where study data are available. | Data availability statement, Lines 518-519 |
| Declaration of interests | [#21a](https://www.goodreports.org/reporting-checklists/arrive2/info/#21a) | Declare any potential conflicts of interest, including financial and nonfinancial. If none exist, this should be stated. | Conflict of interest  Lines: 533-534 |
| Declaration of interests | [#21b](https://www.goodreports.org/reporting-checklists/arrive2/info/#21b) | List all funding sources (including grant identifier) and the role of the funder(s) in the design, analysis, and reporting of the study. | Funding statement  Lines: 537-540 |
