# Supplementary figures and images for: Growth Performance, Blood Chemistry, and Intestinal Bacterial Community of Florida Pompano (Trachinotus carolinus) Fed Different Levels of Corn Fermented Protein and Yeast Diets
Source: Aquac Nutr. 2025 Aug 22;2025:8872997. doi: 10.1155/anu/8872997 (PMC12396910; doi:10.1155/anu/8872997)

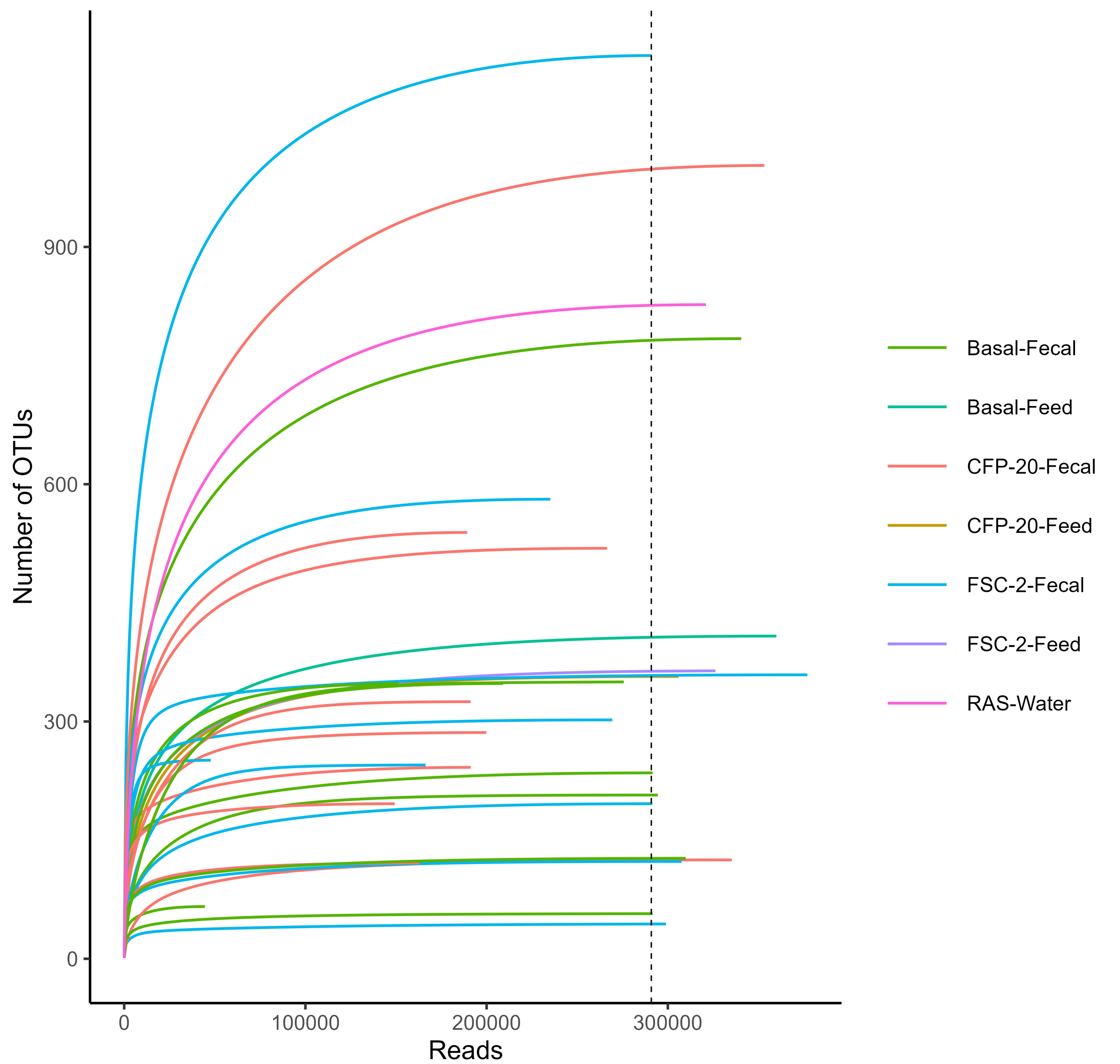

Supplement: Supporting Information 2 — Figure S1. Rarefaction curve sequences showing the microbial community complexities and reads per sample following normalization. CFP-20, corn fermented protein with 20% supplemented diet; FSC-2, Saccharomyces cerevisiae fermentation product 2% supplemented diet; and OTUs, operational taxonomic units. [file 8872997.f2.pdf]
